# Photovoltaic power interval prediction with conditional error dependency using Bayesian optimized deep learning

**DOI:** 10.1038/s41598-025-19602-6

**Published:** 2025-12-13

**Authors:** Yixuan Chen, Xinggang Wang, Run Huang, Guangzeng You

**Affiliations:** Power Grid Planning and Construction Research Center, Yunnan Power Grid Company, Kunming, 650041 China

**Keywords:** CNN-BiLSTM-attention, Conditional dependence of prediction errors, Interval prediction, K-shape, ABKDE, Solar energy, Electrical and electronic engineering

## Abstract

Accurate photovoltaic (PV) power forecasting serves as a critical foundation for economic dispatch and reliable grid operation. To address the inherent uncertainty in PV power generation, this study proposes a short-term PV power interval prediction method based on Bayesian-optimized CNN-BiLSTM-attention (BO-CNN-BiLSTM-attention) that accounts for conditional dependencies in prediction errors. The methodology comprises three main stages: first, PV output data undergoes preprocessing and feature selection. Second, a Bayesian-optimized CNN-BiLSTM-attention model achieves high-precision point forecasting for target time periods. Finally, the K-shape time series clustering algorithm matches point predictions with temporally similar historical data, while adaptive bandwidth kernel density estimation models the probability distribution of prediction errors from similar patterns, thereby enabling interval prediction. Experimental validation on a photovoltaic plant in Xinjiang, China demonstrates that the proposed method achieves superior prediction accuracy compared to various single and ensemble forecasting models, while outperforming multiple interval construction approaches in terms of prediction effectiveness.

## Introduction

With the proposal of the “dual carbon” goals, the construction of new power systems is advancing steadily, and modern power systems are evolving toward a "dual-high" paradigm characterized by high proportions of power electronic equipment applications and renewable energy integration. As one of the most significant forms of renewable energy generation, photovoltaic (PV) power generation has become increasingly critical for grid scheduling, planning, and safe, stable operation^1^. Given that the high volatility and strong randomness of PV output pose substantial challenges to accurate forecasting, an increasing number of researchers have begun attempting to quantify PV output uncertainty through interval prediction, thereby providing more comprehensive PV output forecasting information^2^.

PV output prediction methods are primarily categorized into deterministic prediction and uncertainty prediction^3^. Deterministic prediction methods mainly refer to point prediction, and most studies on PV output prediction have centered on point prediction to date. The main technical approaches can be classified into physical methods, statistical methods, and hybrid prediction methods that combine both approaches.

Physical methods for PV output point prediction are primarily based on numerical weather prediction (NWP) models, satellite imagery, and sky imaging data sources, predicting PV output by simulating atmospheric behavior, solar radiation transmission, and cloud coverage processes. These methods involve detailed modeling of the physical characteristics of PV systems, including solar radiation separation and conversion, electrical characteristics of PV components, and environmental factor influences. For instance, Yuan et al.^4^ proposed a meteorological forecast-based physical prediction model for PV power, achieving meteorological parameter prediction through hierarchical clustering and implementing PV power point prediction combined with an improved incremental conductance method. János et al.^5^ conducted a comprehensive comparative analysis of physical models for PV power prediction, demonstrating that model selection significantly impacts prediction accuracy, with average absolute error differences reaching up to 13% between the most and least accurate models. Physical methods offer advantages in interpretability, small-sample effectiveness, and stability, making them suitable for medium- to long-term prediction. However, they require high-quality meteorological data and exhibit unstable accuracy under extreme weather conditions.

Statistical methods for PV output point prediction are primarily based on historical data, establishing prediction models by analyzing statistical relationships between historical meteorological data and PV power data. These methods mainly include traditional statistical methods, machine learning methods, and currently prevalent deep learning methods: (a) Traditional statistical methods: These include autoregressive integrated moving average (ARIMA) models, seasonal autoregressive integrated moving average (SARIMA) models, and various regression analysis methods. ARIMA models, as typical representatives of Box-Jenkins time series modeling methodology^6^, have been widely applied in PV output point prediction research^7–9^. SARIMA builds upon ARIMA by considering seasonal patterns in data, possessing the capability to capture both short-term and long-term dependencies, enabling more accurate predictions^10^. Regression analysis methods achieve prediction by establishing regression relationships between PV power and meteorological factors, offering advantages of model simplicity and high computational efficiency^11^. However, traditional statistical methods^6–11^ have extremely limited capabilities in handling nonlinearity and non-stationarity in data, making it difficult to effectively capture complex patterns in PV power data. (b) Machine learning methods: These methods overcome some limitations of traditional statistical approaches by learning complex nonlinear relationships from historical data to achieve PV power prediction. Mainstream machine learning methods include support vector machines (SVM)^12^, artificial neural networks (ANN)^13^, extreme learning machines (ELM)^14^, and random forests^15^. These methods can process high-dimensional feature data with strong nonlinear fitting capabilities. Raza et al.^16^ proposed a multivariate neural network (NN) ensemble framework for PV output point prediction by training multiple neural network predictors combined with Bayesian model averaging techniques, providing practical solutions for improving seasonal PV power output prediction accuracy. Liu et al.^17^ integrated three different model types—SVM, MLP, and MARS—through recursive arithmetic averaging, proposing a data-driven ensemble modeling technique with prediction performance significantly superior to individual models. While machine learning methods^12–17^ can utilize large-sample PV power data to achieve relatively accurate PV output prediction, they still have limitations in handling long-term dependencies in time series data and limited prediction effectiveness when facing large-scale, high-dimensional complex datasets. (c) Deep learning methods: The emergence of recurrent neural networks (RNN), long short-term memory networks (LSTM), gated recurrent units (GRU), and convolutional neural networks (CNN) has effectively addressed the limitations of traditional machine learning methods, particularly excelling in handling complex temporal relationships in time series data. Maria et al.^18^ achieved precise hourly PV output prediction based on stacked long short-term memory networks. Sun et al.^19^ proposed a specialized convolutional neural network model called "SUNSET," which effectively addresses uncertainty caused by cloud changes in short-term solar power prediction by fusing sky images and historical PV power data, improving prediction accuracy and reliability. Furthermore, advanced deep learning architectures have demonstrated enhanced capabilities through sophisticated structural innovations. Niu et al.^20^ developed Solar-Mixer, an efficient end-to-end model that integrates anomaly detection with interval-mixing and channel-mixing layers based on multilayer perceptrons, enabling accurate long-sequence time series forecasting while maintaining low computational complexity through pure MLP-based architecture. Deep learning methods can automatically extract features and effectively capture complex nonlinear relationships in data, achieving further accuracy improvements in PV power prediction compared to traditional machine learning methods.

Hybrid prediction methods that integrate multi-domain, multi-disciplinary, or multi-type technical approaches have gained scholars’ attention to fully combine the advantages of different methods and maximize high-precision PV output prediction. These include physics-statistics hybrid methods, deep learning combination methods, and ensemble models. Physics-statistics hybrid methods combine the mechanistic nature of physical models with the flexibility of statistical methods to enhance prediction performance. Mellit et al.^10^ developed a hybrid SARIMA-SVM model for short-term power prediction of small grid-connected PV plants, achieving a normalized root mean square error of only 9.57%, significantly outperforming individual SARIMA or SVM models. Theocharides et al.^21^ proposed a day-ahead PV power prediction method based on machine learning and statistical post-processing, combining numerical weather prediction (NWP) outputs with historical observational data to effectively improve prediction accuracy. Deep learning combination methods emphasize fusing advantages of different deep learning models to enhance prediction performance, with CNN-LSTM hybrid architectures being typical representatives. Chai et al.^22^ combined CNN’s spatial feature extraction capabilities with LSTM’s nonlinear temporal dependency fitting abilities, proposing a CNN-LSTM method for PV output point prediction while introducing correntropy criteria to handle data contamination, improving model robustness for simultaneous PV power prediction across multiple regions and time periods. Li et al.^23^ proposed a temporal convolutional network (TCN)-based hybrid prediction framework for utility-scale PV forecasting. Niu et al.^24^ proposed a mid-term PV forecasting system employing a 'De-Trend First, Attend Next’ strategy that separates trend and seasonal components before applying encoder-decoder structures with temporal convolution and attention mechanisms, achieving 73% improvement in mean squared error through component-specific modeling. The same research team also introduced an innovative 'Amplify Seasonality, Prioritize Meteorological’ approach that leverages dual-layer hierarchical attention mechanisms to strengthen connections between meteorological features and seasonal components while protecting trend components from short-term meteorological fluctuations, demonstrating over 10% improvement in Mean Absolute Error^25^. Furthermore, advanced ensemble methodologies have enhanced prediction robustness across diverse operational conditions. Cao et al.^26^ developed an ultra-short-term PV forecasting framework based on Stacking ensemble algorithm (StAB) that integrates correlation-guided fast Fourier transform decomposition with multi-model optimization, achieving superior generalization performance across different distributed PV systems. Overall, these hybrid methods can effectively reduce point prediction errors of individual models, improving prediction stability and reliability, making them optimal choices for current PV output point prediction. Overall, these hybrid methods can effectively reduce point prediction errors of individual models, improving prediction stability and reliability, making them optimal choices for current PV output point prediction.

The above methods are all point prediction approaches that offer the advantage of intuitive prediction results; however, they provide very limited prediction information and cannot reflect global uncertainty. Interval prediction methods represent a viable solution to this challenge. Interval prediction methods obtain upper and lower bounds of PV output at given confidence levels, effectively quantifying PV output uncertainty and providing richer prediction information. Existing interval prediction methods can be specifically categorized into two types:

The first type is direct interval prediction, where prediction methods can directly output upper and lower bounds for interval prediction without relying on deterministic prediction results. Literature^27^ proposed an ensemble method (ELUBE) based on ELM and Lower upper bound estimation (LUBE) to achieve direct interval prediction of PV power. To improve prediction interval quality, the authors employed three different activation functions—sigmoid, radial basis, and sine functions—to train multiple ELUBE models, combining selected high-performance models through weighted averaging methods. Zhao et al.^28^ proposed an efficient probabilistic interval prediction method for PV output by improving Bayesian neural networks, introducing probabilistic representation weights, t-distributed stochastic neighbor embedding algorithm for dimensionality reduction, and fully connected and convolutional neural networks, achieving superior accuracy and reliability compared to traditional models.

The second type is indirect interval prediction, which constructs confidence intervals for PV output through other methods based on point prediction results. Han et al.^29^ analyzed seasonal distribution characteristics of PV power, utilized seasonal multi-models of Extreme Learning Machines (ELM) for deterministic prediction, then employed kernel density estimation to fit deterministic prediction errors, proposing an indirect interval prediction method for PV power combining multi-models and non-parametric estimation. Literature^30^ proposed a PV power point prediction method based on hybrid intelligent models, optimized using wavelet transforms and radial basis neural networks, achieving indirect and direct interval prediction of PV power through Bootstrap and quantile regression methods, respectively, further expanding neural network applications in time series data learning and providing new insights for PV energy prediction. Yang et al.^31^ employed commonly used recurrent neural networks (LSTM, GRU) to achieve PV output point prediction, comprehensively corrected point prediction results considering temporal and spatial dependencies of PV power, and finally implemented indirect interval prediction of PV output using kernel density estimation methods based on conditional dependencies of PV output errors. However, due to their relatively single point prediction approach, the point prediction effectiveness still requires improvement. The so-called conditional dependency of PV output errors refers to the fact that the probability distribution of PV output is related to factors such as prediction time scales and prediction models. Research addressing this includes conventional methods^32,33^, methods considering spatial dependencies^34^, and methods for statistical analysis of errors within the same time periods^35^.

In summary, this paper proposes a short-term PV output interval prediction method based on Bayesian-optimized CNN-BiLSTM-Attention (BO-CNN-BiLSTM-Attention) that accounts for conditional dependencies of prediction errors. The methodology comprises the following steps: First, PV power data undergo preprocessing operations including missing value imputation, outlier replacement, and nighttime data removal. Second, the MIC method analyzes correlations between PV power data and multi-dimensional features for feature selection engineering. Third, a Bayesian algorithm-optimized CNN-BiLSTM-Attention method achieves high-precision point prediction of PV output for target time periods. Finally, the K-shape clustering algorithm matches historical optimal similar days for target prediction periods, and adaptive bandwidth kernel density estimation (ABKDE) performs kernel density estimation on error sample sets of similar days, thereby achieving interval prediction of PV output. Experimental results demonstrate that the proposed method achieves higher prediction accuracy compared to various benchmark prediction models and combined prediction models.

The main contributions of the proposed method in this paper are as follows:

1. Introduced the Adaptive Bandwidth Kernel Density Estimation (ABKDE) method for PV power prediction error modeling that dynamically adjusts bandwidth parameters according to local sample density characteristics. Unlike conventional fixed-bandwidth approaches, ABKDE employs iterative optimization to determine optimal local bandwidth factors, providing enhanced density estimation capabilities for unevenly distributed prediction error datasets.
2. Integrated the K-shape time series clustering algorithm to establish a conditional error dependency-aware interval prediction architecture. This approach identifies historical similar days based on temporal shape patterns rather than numerical value similarity, enabling weather-specific error distribution modeling that recognizes the relationship between meteorological conditions and prediction error characteristics.
3. Designed a hierarchical CNN-BiLSTM-Attention framework that combines multi-scale convolutional feature extraction with bidirectional temporal dependency modeling. The architecture employs progressive CNN layers with kernel sizes (1, 3, 5) for hierarchical feature learning, BiLSTM networks for capturing forward and backward temporal relationships, and temporal pattern attention mechanisms for adaptive time-step weighting.
4. Established a systematic interval prediction methodology that integrates shape-based clustering with adaptive density estimation to address prediction error conditional dependency. This comprehensive approach provides a novel framework for uncertainty quantification in PV forecasting by systematically accounting for the non-uniform distribution of prediction errors across different environmental and temporal conditions.

## Theoretical algorithm

### Maximal information coefficient

Feature selection engineering is necessary to avoid overfitting of the prediction model and reduce the computational burden on the model. Given that photovoltaic power exhibits strong nonlinear relationships with multidimensional input features^36^, and considering the limitations of commonly used linear analysis methods such as Pearson correlation analysis, this study adopts the Maximal Information Coefficient (MIC) to analyze the correlations between photovoltaic power and multidimensional input features and to perform feature selection engineering. MIC is a statistical method that can capture both linear and nonlinear relationships while being relatively insensitive to noise in the data^37^. Its core principle involves locally maximizing the mutual information between two variables to identify the strongest relationship. The calculation process consists of three main steps: First, the two-dimensional data space is divided into *a* and *b* intervals in the *X* and *Y* directions, respectively, forming *ab* grids. Second, the mutual information between the two variables is calculated within each grid. Finally, the maximum mutual information value is identified across all grids and used as the MIC value. The mutual information between two variables *x* and *y* is shown in Eq. (1), and the MIC calculation formula is presented in Eq. (2).

1 $$I(x,y) = \sum\limits_{x \in X} {\sum\limits_{y \in Y} {p(x,y)} } \log_{2} \left( {\frac{p(x,y)}{{p\left( x \right)p\left( y \right)}}} \right)$$

2 $$MIC(X,Y) = \max_{ab < C(n)} \left\{ {M(C)_{a,b} } \right\}$$

3 $$M(C)_{x,y} = \frac{{I\left( {C,x,y} \right)}}{{\log_{2} \min \left\{ {a,b} \right\}}}$$

where: *n* is the total number of samples; *C*(*n*) is a function that represents the upper limit of meshing, generally taken as *C*(*n*) = *n*^0.6^^38^.

### Convolutional neural network

**Fig. 1:**
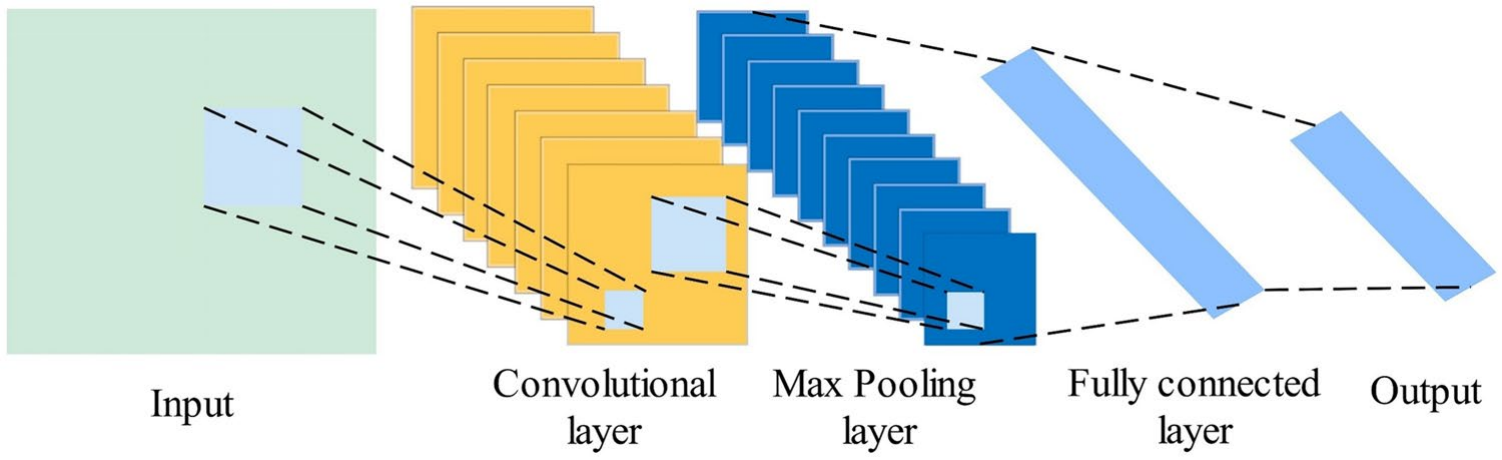
CNN network structure diagram

CNNs are widely-used deep learning models for processing grid-structured data and time series feature extraction^39^. As shown in Fig. 1, CNNs comprise input layers, convolutional layers, pooling layers, and fully connected layers. Data enters through the input layer and undergoes convolution operations via Eq. (4) to capture hierarchical structures and learn local features.

4 $$y_{i,j} = f\left( {\sum\limits_{m = - k/2}^{k/2} {\sum\limits_{n = k/2}^{k/2} {w_{m,n} x_{i + m,j + n} + w_{b} } } } \right)$$

where *x* represents input data; *y* denotes convolutional layer output (extracted features); *w* is the convolution kernel; *k* represents kernel size; *i* and *j* indicate output positions; *m* and *n* represent kernel positions; *w*_*b*_ denotes bias terms; *f* is the activation function.

Pooling layers, positioned between consecutive convolutional layers, reduce spatial dimensions of extracted features while preserving essential information. This study employs max pooling, which selects maximum values from each region as representatives via Eq. (5).

5 $$\begin{aligned} & \max {\text{plooing}} [i,j] = \\ & \quad \quad \max (x[i * s_{h} :(i * s_{h} + h),j * s_{w} :(j * s_{w} + w)]) \\ \end{aligned}$$

where maxpooling represents the pooling result; *x* denotes the input matrix; *s*_*h*_ and *s*_*w*_ represent vertical and horizontal strides respectively; *h* and *w* represent pooling window dimensions.

Fully connected layers implement forward propagation via Eq. (6), flattening convolutional and pooling outputs into vectors for classification or regression tasks.

6 $$y_{k} = f\left(\sum\limits_{i = 1}^{t} {W_{k,i} x_{i} + b_{k} } \right)$$

where *y*_*k*_ represents the *k*-th neuron output; *x*i denotes the *i*-th element of the flattened feature vector; *W*_*k*,*i*_ represents connection weights from the *i*-th input to *k*-th output neuron; *b*_*k*_ denotes the *k*-th output neuron bias; *t* represents input feature vector dimensionality.

### Bidirectional long short-term memory network

**Fig. 2:**
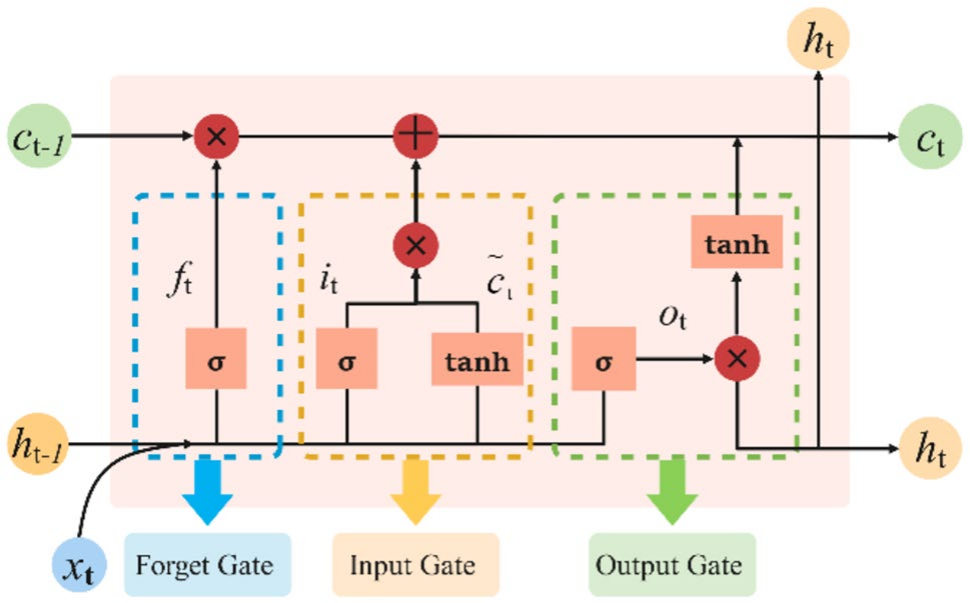
LSTM network structure diagram

BiLSTM builds upon LSTM improvements, with LSTM architecture shown in Fig. 2. LSTM’s core concept involves memory cells and gating mechanisms for adaptive information reading, writing, and forgetting to address long-term dependencies in sequence processing. As illustrated in Fig. 2, the forget gate *f*_*t*_ controls information retention from previous cell state *c*_*t* − 1_ through sigmoid activation, determining which historical information to discard. The input gate *i*_*t*_ regulates current input importance, collaborating with candidate values $\widetilde{c}_{t}$ to determine new information storage. Candidate values $\widetilde{c}_{t}$ generate new candidate information via tanh activation. Cell state *c*_*t*_ maintains long-term memory through interaction with previous state *c*_*t-*1_ and forget gate *ft*, while incorporating short-term information via *i*_*t*_·$\tilde{c}$_*t*_. The output gate *o*_*t*_ controls which cell state information outputs to hidden state *h*_*t*_, enabling selective information transmission. This coordinated gating mechanism enables LSTM to effectively maintain and transmit critical information across long sequences, overcoming traditional RNN gradient vanishing problems. LSTM computations follow Eq. (7).

7 $$\begin{gathered} i_{t} = \sigma (W_{i} x_{t} + U_{i} h_{t - 1} + b_{i} ) \hfill \\ f_{t} = \sigma (W_{f} x_{t} + U_{f} h_{t - 1} + b_{f} ) \hfill \\ o_{t} = \sigma (W_{o} x_{t} + U_{o} h_{t - 1} + b_{o} ) \hfill \\ \widetilde{c}_{t} = \tanh (W_{c} x_{t} + U_{c} h_{t - 1} + b_{c} ) \hfill \\ c_{t} = i_{t} \cdot \widetilde{c}_{t} + f_{t} \cdot c_{t - 1} \hfill \\ h_{t} = o_{t} \cdot \tanh (c_{t} ) \hfill \\ \end{gathered}$$

where *x*_*t*_ represents input at time *t*; *h*_*t* − 1_ denotes hidden state at time *t* − 1; *i*_*t*_ represents input gate state; *c*_*t*_ denotes cell state; *σ* represents sigmoid activation; tanh denotes hyperbolic tangent activation; ***W***_*f*_, ***W***_*i*_, ***W***_***c***_, ***W***_*o*_ and ***U***_*f*_, ***U***_*i*_, ***U***_*c*_, ***U***_*o*_ are weight matrices for forget, input, candidate, and output gates multiplying *x*_*t*_ and *h*_*t-*1_ respectively; *b*_*f*_, *b*_*i*_, *b*_*c*_, *b*_*o*_ are corresponding bias terms.

**Fig. 3:**
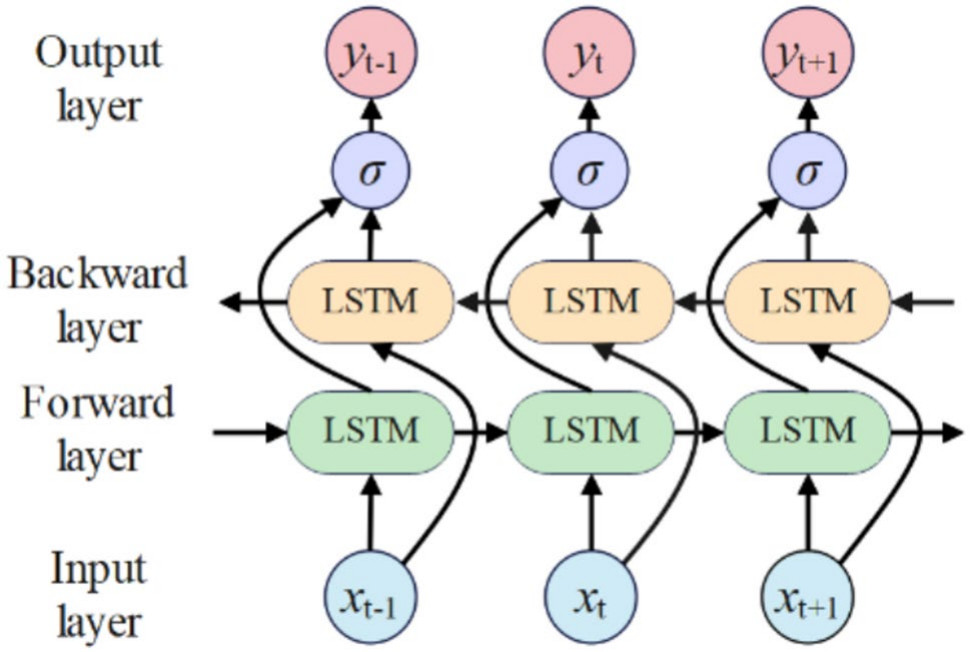
BiLSTM network structure diagram

Unlike traditional LSTM, BiLSTM comprises forward and backward LSTM components processing bidirectional temporal information, better capturing contextual information in time series data with bidirectional dependency capabilities while retaining LSTM’s gradient vanishing mitigation^40^. BiLSTM structure appears in Fig. 3.

As shown in Fig. 3, forward LSTM processes from sequence start, receiving current input *x*_*t*_ and previous forward hidden state *hf t-*1, updating forward hidden states and memory cells via Eq. (8) to obtain *hf t*.

8 $$h_{t}^{f} = {\text{LSTM}} (x_{t} ,h_{t - 1}^{f} )$$

where LSTM(·) represents LSTM unit computations following Eq. (7) for hidden state updates.

Simultaneously, backward LSTM processes from sequence end, receiving current input *x*_*t*_ and subsequent backward hidden state *hb t-1*, updating backward hidden states and memory cells via Eq. (9) to obtain *hb t*.

9 $$h_{t}^{b} = {\text{LSTM}} (x_{t} ,h_{t - 1}^{b} )$$

Finally, Eq. (10) concatenates forward and backward hidden state sequences temporally to produce final output sequence *y*_*t*_.

10 $$y_{t} = \sigma \left( {{\mathbf{W}}_{y} \cdot \left[ {h_{t}^{f} ,h_{t}^{b} } \right] + b_{y} } \right)$$

where ***W***_*y*_ represents weight matrices for linear combination of hidden layer states; *b*_*y*_ denotes bias terms.

### Attention mechanism

While CNN-BiLSTM models can capture nonlinear relationships between PV power data and multi-dimensional input features, prediction performance may suffer from increasing time series lengths. Attention mechanisms simulate biological attention, analogous to humans focusing on specific regions due to limited visual resources, successfully applied in machine translation and time series prediction^41^. Applying temporal attention mechanisms to CNN-BiLSTM outputs enables adaptive selection of relevant historical time series state information, obtaining corresponding temporal sequence hidden state weights and determining temporal state influence on current PV power output, thereby extracting critical temporal information to improve prediction performance. The temporal attention mechanism operates as follows:

Encode BiLSTM’s final hidden layer output *h* = {*h*_1_,*h*_2_,*h*_*t*_,···,*h*_*T*_} to obtain query vector *q*; then employ additive attention to compute historical temporal state importance for current output. The attention scoring formula follows Eq. (11).

11 $$Score\left( {h_{t} } \right){ = }\tanh (W_{{\text{s}}} h_{t} + b_{s} )$$

where Score(*h*_*t*_) represents similarity scores indicating correlation between historical temporal state *h*_*t*_ and output; ***W***_*s*_ denotes weight matrices; *b*_*s*_ represents bias terms.

Subsequently, softmax normalization produces input vector weights *a*_*t*_ via Eq. (12).

12 $$a_{t} = {\text{softmax}} (S{\text{core}}\left( {h_{t} } \right)) = \frac{{\exp \left( {S{\text{core}}\left( {h_{t} } \right)} \right)}}{{\sum\limits_{i = 1}^{n} {\left( {S{\text{core}}\left( {h_{t} } \right)} \right)} }}$$

Based on step (1) attention weight calculations for historical temporal states, weighted averaging produces final attention-optimized output *h* via Eq. (13).

13 $$h = \sum\limits_{t = 1}^{T} {a_{t} h_{t} }$$

### K-shape clustering

The K-shape clustering algorithm is specifically designed for time series data clustering. Unlike traditional clustering algorithms such as K-means, K-means++, and DBSCAN, which rely primarily on numerical values, K-shape identifies the underlying “shape” or pattern of time series data. This capability stems from K-shape’s ability to perform normalization, horizontal translation, and vertical stretching operations on the clustered objects. Consequently, K-shape is particularly well-suited for handling time series data that exhibit obvious dynamic changes and nonlinear characteristics^42^. The specific clustering steps of K-shape are as follows:

Measure the distance between time series data. Unlike traditional clustering algorithms, K-shape uses a shape-based distance (SBD) based on slope, intercept and normalized cross correlation coefficient (*NCC*_*C*_) to measure the distance between time series data. The SBD-based distance metric ensures that the scale and offset of time series data do not affect the distance calculation accuracy. For two time series *x* = (*x*_1_,* x*_2_, ···, *x*_n_) and *y* = (*y*_1_,* y*_2_, ···, *y*_n_), the distance metric formula is as follows:

14 $$SBD({\varvec{x}},{\varvec{y}}) = 1 - \max (NCC_{C} ({\varvec{x}},{\varvec{y}}))$$

15 $$NCC_{C} ({\varvec{x}},{\varvec{y}}) = \frac{{CC_{\omega } ({\varvec{x}},{\varvec{y}})}}{{\sqrt {R_{0} ({\varvec{x}},{\varvec{x}}) \cdot R_{0} ({\varvec{y}},{\varvec{y}})} }}$$

16 $$R_{\omega - m} ({\mathbf{x}},{\mathbf{y}}) = \left\{ {\begin{array}{*{20}c} {\sum\limits_{l = 1}^{2m - \omega } {{\mathbf{x}}_{l} + (\omega - m) \cdot {\mathbf{y}}_{l} ,} } & {\omega - m \ge 0} \\ {R_{ - (\omega - m)} ({\mathbf{y}},{\mathbf{x}}),} & {\omega - m < 0} \\ \end{array} } \right.$$

17 $$CC_{\omega } ({\mathbf{x}},{\mathbf{y}}) = R_{\omega - m} ({\mathbf{x}},{\mathbf{y}})$$

where: *CC*_*ω*_ (***x***,***y***) represents the inter-correlation sequence of length 2*m* − 1, *ω* ∈ {1,2, ···,2*m* − 1}. The value of SBD(x,y) is in the range of [0, 2], and the smaller the value, the smaller the degree of dissimilarity between the two time series data.

Extract representative features from time series data. K-shape calculates cluster centroids and assigns each sequence to distinct cluster groups based on their distance relationships to these centroids. The centroid computation process resembles an optimization problem, where the algorithm seeks to identify the cluster centroid ***μ***_i_^*^ that maximizes the squared similarities to all constituent time series sequences. The process of calculating the cluster center is similar to the optimal value problem, and the goal is to find the cluster center corresponding to the maximum quadratic similarity, which is calculated as shown in Eq. (18).

18 $${{\varvec{\upmu}}}_{i}^{ * } { = }\mathop {\arg \max }\limits_{{{{\varvec{\upmu}}}_{i} }} \sum\limits_{x \in Ci} {(NCC_{c} ({\mathbf{x}},{{\varvec{\upmu}}}_{i} ))^{2} }$$

where *C*_*i*_ represents the* i*-*th* homoscedastic cluster class. ***μ***_*i*_ represents the initial center of mass of the* i*-*th* cluster class.

**Fig. 4:**
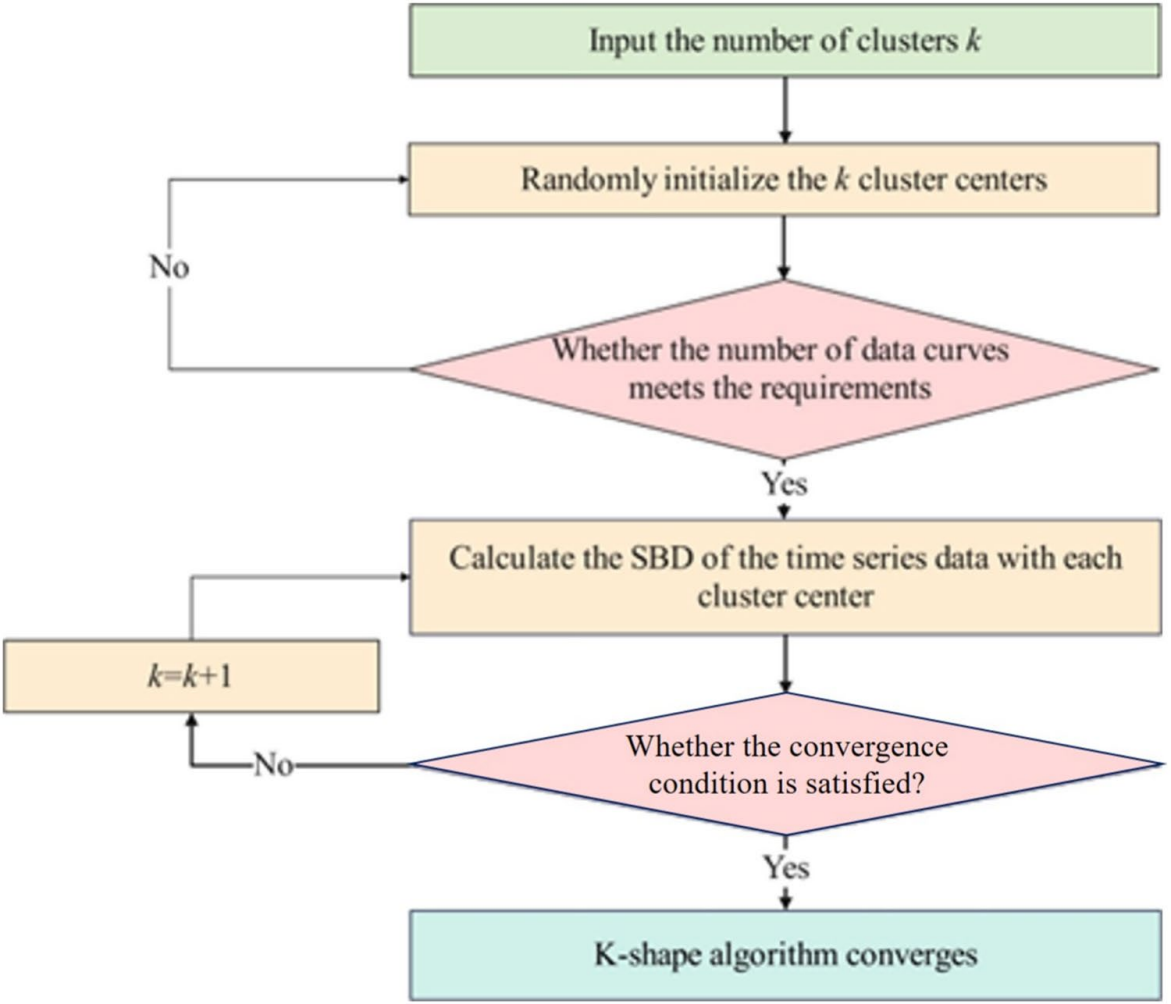
K-shape algorithm flowchart

Perform shape-based clustering of time series. The algorithm implements shape-based clustering through the aforementioned distance measurement and feature extraction procedures. Initially, cluster centroids are randomly initialized, followed by iterative computation of each cluster’s centroid based on the optimization framework described in step 2). The algorithm employs SBD metrics to evaluate clustering effectiveness and refine cluster assignments. The iterative process continues until convergence is achieved, which occurs when cluster assignments remain unchanged between consecutive iterations or when the maximum number of iterations is reached (typically 100 iterations), following the standard k-means convergence paradigm. The specific flow of K-shape clustering is shown in Fig. 4.

### Adaptive bandwidth kernel density estimation

Kernel density estimation (KDE) is a nonparametric method for estimating the probability density function of data, which has the advantages of high adaptability and flexibility, and its main principle is to estimate the density of a sample with the help of a sliding window. Considering that a fixed window bandwidth may not be well adapted to the estimation of the probability density of the PV output prediction error, this paper decides to use ABKDE to realize the kernel density estimation of the PV output prediction error. Adaptive bandwidth kernel density estimation allows the bandwidth to vary according to local sample density and determines optimal local bandwidth through iterative optimization. This approach provides particularly effective density estimation for unevenly distributed samples^43^. Typical density estimation functions based on Gaussian kernel functions are shown in Eqs. (19) and (20). The formulas for iteratively solving the optimal local bandwidth are shown in Eqs. (21)–(23).

19 $$\sigma_{K} \left( {\xi_{0} } \right) = \frac{1}{N}\sum\limits_{i = 1}^{N} {K(\xi_{i} - \xi_{0} ;\mu )}$$

20 $$K(\xi_{i} - \xi_{0} ;\mu ) = \frac{1}{{\sqrt {2\pi } \mu }}\exp \left( { - \frac{1}{2}\left( {\frac{{\xi_{i} - \xi_{0} }}{\mu }} \right)^{2} } \right)$$

21 $$\sigma_{K,k} (\xi_{0} ) = \left\{ \begin{gathered} \frac{1}{n}\sum\limits_{i = 1}^{n} {K(\xi_{i} - \xi_{0} ;\mu ),\begin{array}{*{20}c} {k = 0} \\ \end{array} } \hfill \\ \frac{1}{n}\sum\limits_{i = 1}^{n} {K(\xi_{i} - \xi_{0} ;\mu \lambda_{i,k} ),\begin{array}{*{20}c} {k \ge 1} \\ \end{array} } \hfill \\ \end{gathered} \right.$$

22 $$\log g_{k} = \frac{{\sum\limits_{i = 1}^{n} {\log \sigma_{K,k} (\xi_{i} )} }}{n}$$

23 $$\lambda_{i,k + 1} = \left( {\frac{{g_{k} }}{{\sigma_{K,k} \left( {\xi_{i} } \right)}}} \right)^{\alpha }$$

where *σ*_*K*_(*ξ*_0_) represents the kernel density at* ξ*_0_ of the sample to be estimated. *K*(·) represents the kernel function.* ξ*_*i*_ represents the *ith* sample, and* μ* is the bandwidth of the kernel density estimation, whose value is relatively smooth when the kernel density distribution is large, and relatively steep when the kernel density distribution is small.* λ*_*i*,*k*+1_ represents the local bandwidth factor obtained from the *kth* iteration, and *K* represents the number of iterative solution times.* σ*_*K*,*k*_(*ξ*_0_) is the kernel density at* ξ*_0_ obtained from the *k*th iteration. g represents the kernel density normalization factor obtained from the *k*th iteration. *α* is the sensitivity factor, which indicates the sensitivity of local bandwidth to the sparsity of the sample distribution. σ_*k*_(*ξ*) is the kernel density at *ξ* obtained in the kth iteration. *g*_*k*_ represents the kernel density normalization factor obtained in the *Kth* iteration.* α* is the sensitivity factor, which indicates the sensitivity of the local bandwidth to the sparsity of the sample distribution, and if* α* is 0, then the ABKDE degenerates to a fixed-bandwidth kernel density estimation.

### BO-CNN-BILSTM-attention-based short-term PV power interval prediction method considering conditional dependence of prediction errors

This study integrates deep learning algorithms, time series clustering algorithms, and statistical probabilistic density analysis methods, proposing a short-term PV output interval prediction method based on BO-CNN-BiLSTM-Attention considering prediction error conditional dependency. First, MIC analysis comprehensively examines correlations between input features and PV power data for feature selection. Then, BO-CNN-BiLSTM-Attention hybrid neural networks achieve high-precision point prediction, establishing foundations for subsequent interval prediction. Based on point predictions, K-shape algorithms match historical similar-day PV power data for target prediction periods, while ABKDE fits prediction errors from historical similar days to construct prediction intervals.

**Fig. 5:**
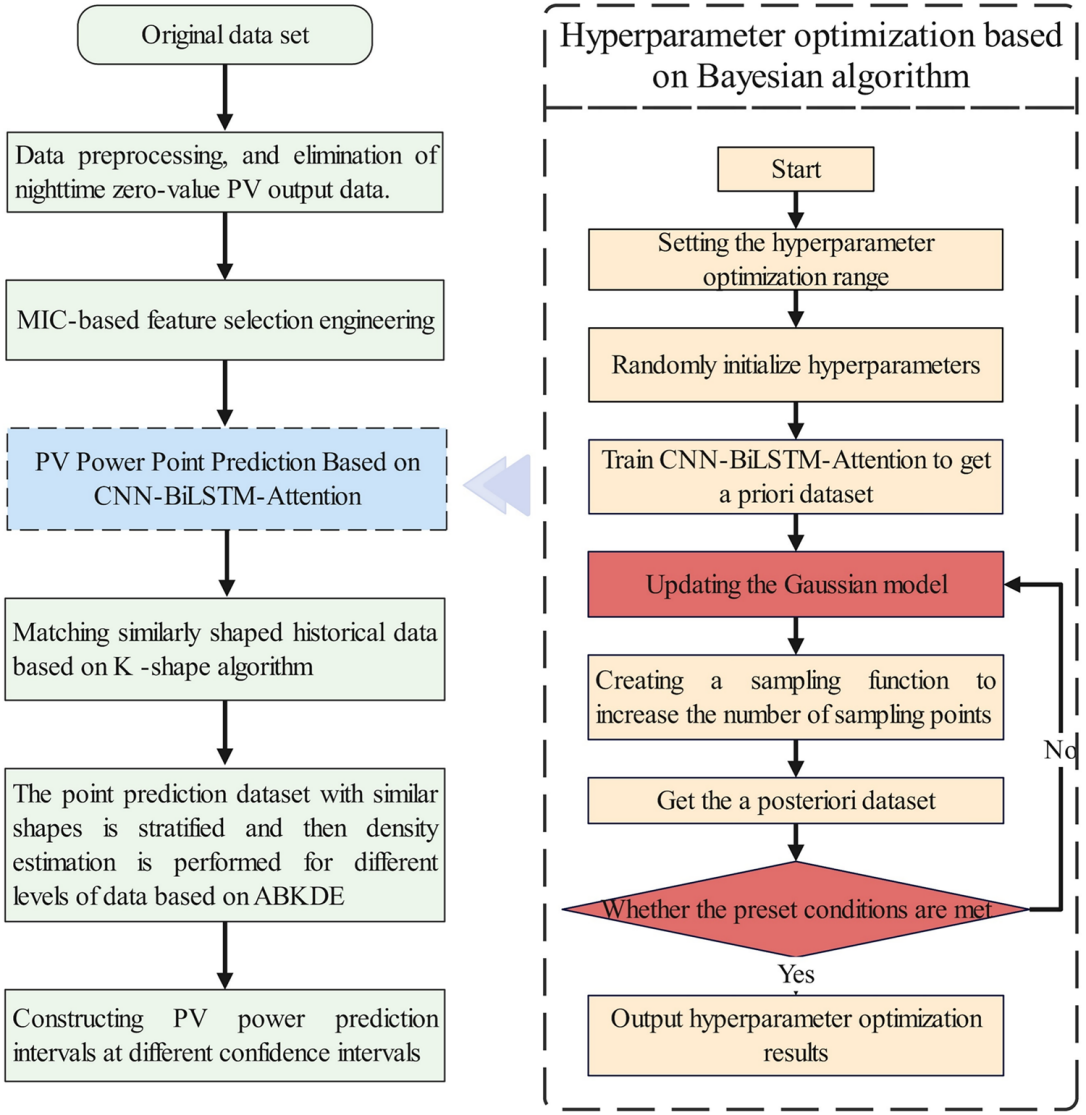
Overall framework of the proposed methodology.

Considering numerous hyperparameters in hybrid neural networks directly impact prediction performance, hyperparameter optimization becomes essential. For neural network hyperparameter optimization, Bayesian algorithms efficiently explore and exploit hyperparameter spaces with adaptive search strategies, providing reliable optimization results for complex black-box functions. These algorithms utilize prior evaluation results to update posterior probability distributions of objective functions, guiding subsequent sample selection to identify superior hyperparameter settings efficiently. Detailed Bayesian optimization algorithms appear in literature^44^. Figure 5 presents the integrated framework combining Bayesian optimization workflows with the proposed methodology.

**Fig. 6:**
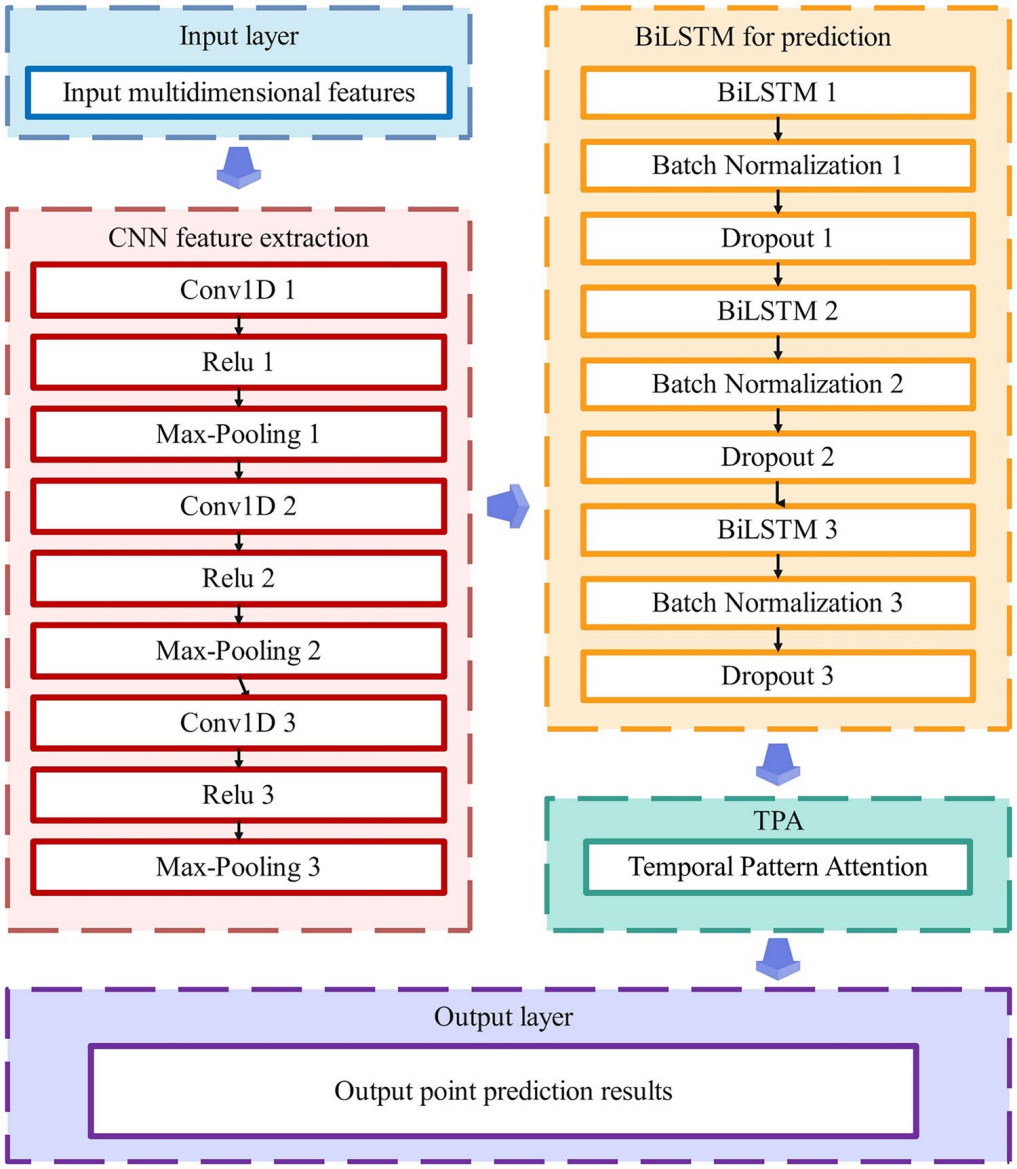
CNN-BiLSTM-attention overall framework diagram.

Considering the strong volatility and high stochasticity of PV power, a single recurrent neural network prediction model, such as RNN, GRU, LSTM, BiLSTM, etc., may not be able to fit the extremely complex nonlinear relationship between PV power and each input feature well. Therefore, in this paper, three-layer CNN is used to process the input data and extract its local spatial features, and the convolution kernel sizes of the three-layer CNN are set to 1, 3, and 5 sequentially to fully extract the time series features at different scales layer by layer. Then, the long-term dependence between the PV power and the input features is fully captured based on the three-layer BiLSTM to realize the nonlinear fitting. Finally, Temporal Pattern Attention mechanism is employed to focus on identifying important temporal patterns in the CNN-BiLSTM output data and dynamically weighting different time steps to further improve the prediction accuracy of the model. The overall framework of CNN-BiLSTM-Attention proposed in this paper is shown in Fig. 6.

## Case study

In this paper, the PV power dataset and the corresponding NWP dataset of a PV power plant with a capacity of 50 MW in Xinjiang, China, are selected for the study. The time span of the dataset is February 28, 2019–May 31, 2019, and the sampling interval is 15 min, with a total of 8832 sets of data, and the dataset has no missing data.

### Data pre-processing

The original PV output data with zero values during nighttime periods were excluded, and only valid PV output data from 56 sampling points during 7:00–20:45 were retained. Thus, the total sample size is reduced from 8832 to 5152. Meanwhile, considering the magnitude differences between historical PV power data and multidimensional features, which could affect model prediction accuracy if used directly as input features, the Min–Max normalization method is applied to linearly scale the original data as follows.

24 $$x^{\prime } = \frac{{x - x_{\min } }}{{x_{\max } - x_{\min } }}$$

where: *x'* is the normalized data; *x* is the original data; *x*_max_, *x*_min_ are the maximum and minimum values in the original data.

### Feature selection

MIC was used to analyze the magnitude of correlation between the PV power data and each of the input features, and all the features and their correlation magnitudes are shown in Table 1. As seen in Table 1, the three meteorological factors directly related to solar irradiance, global horizontal irradiance, direct normal irradiance, and diffuse horizontal irradiance, have the greatest impact on PV power, all above 0.7. This is followed by the three characteristics: historical data of PV power one day, two days, and one week ago. It is worth noting that the module temperature of the PV power generation device also has a large impact on the PV power, which is close to 0.5. Only the ambient temperature and relative humidity have a smaller impact on the PV power, and the MIC value after retaining two decimals is only 0.17. In summary, in order to give full play to the feature extraction ability of CNN as much as possible, this paper only excludes the two features of ambient temperature and relative humidity, which have very little correlation with the PV power, and the remaining eight features are all used as the input features for PV power prediction.

**Table 1:** Optional set of input features.

Feature	Correlation
Component temperature (°C)	0.48
Ambient temperature (°C)	0.17
Atmospheric pressure (hPa)	0.38
Relative humidity (%)	0.17
Global horizontal irradiance (W/m)^2^	0.84
Direct normal irradiance (W/m)^2^	0.84
Diffuse horizontal irradiance (W/m)^2^	0.72
PV power a week ago (MW)	0.64
PV power two day ago (MW)	0.65
PV power one day ago (MW)	0.68

### Bayesian-based hyperparameter optimization

The CNN-BiLSTM-Attention combination prediction model can achieve highly accurate photovoltaic power point prediction. However, the model’s performance is critically dependent on proper hyperparameter configuration. If the high-dimensional hyperparameters are not reasonably set, the prediction effectiveness will be significantly compromised. Unfortunately, commonly used optimization approaches such as grid search methods and other intelligent optimization algorithms consume considerable computational time and resources. Therefore, this paper adopts the Bayesian optimization algorithm based on mathematical statistics to realize the efficient optimization of high-dimensional hyperparameters. This study implements Bayesian optimization using the Tree-structured Parzen Estimator (TPE) algorithm within the Optuna package. The optimization process is configured with 200 search iterations and incorporates a pruning strategy to enhance computational efficiency. Additionally, a patience parameter of 50 is implemented as an early stopping mechanism. Specifically, if 50 consecutive training iterations fail to improve the objective function value, the optimization process terminates early to prevent unnecessary computational overhead. The optimization range of hyperparameters and optimization results are shown in Table 2.

**Table 2:** Scope and results of CNN-BiLSTM-attention hyperparameter search based on Bayesian algorithm.

Optimized component	Hyperparameters	Range	Result
CNN	*n_channels 1*	[8,512]	150
	*n_channels 2*	[8,512]	382
	*n_channels 3*	[8,512]	210
	*pool_size1*	[1,5]	1
	*pool_size1*	[1,5]	2
	*pool_size1*	[1,5]	1
BiLSTM	*hidden_size1*	[16,512]	226
	*hidden_size1*	[16,512]	278
	*hidden_size1*	[16,512]	272
	*dropout_rate1*	[0.1,0.5]	0.2
	*dropout_rate1*	[0.1,0.5]	0.2
	*dropout_rate1*	[0.1,0.5]	0.3
*Learning rate*		[1e−5, 0.5]	2e−3

### Indicators for the assessment of projected results

Indicators for evaluating the results of point forecasting

In order to accurately assess the accuracy of the model in this paper, the mean absolute error (MAE) and root mean square error (RMSE) were selected to evaluate the point prediction results. The calculation formula of each assessment index is as follows:

25 $$MAE{ = }\frac{{1}}{n}\sum\limits_{i - 1}^{n} {\left| {\hat{y}_{i} - y_{i} } \right|}$$

26 $$RMSE{ = }\sqrt {\frac{{1}}{n}\sum\limits_{i - 1}^{n} {\left( {\hat{y}_{i} - y_{i} } \right)^{2} } }$$

where: both RMSE and MAE are in MW. *n* represents the number of test set data. ${\widehat{y}}_{i}$ is the predicted value of the *ith* prediction sample. *y*_*i*_ is the true value of the *ith* test sample.

Indicators for evaluating the results of interval forecasting

The prediction interval coverage *E*_PICP_, the average width of the prediction interval *E*_*PINAW*_, and the composite indicator *E*_CWC_ are selected as the evaluation indexes. The larger the *E*_PIC*P*_, the smaller the *E*_PINAW_, the lower the composite indicator *E*_CWC_, the better the prediction effect. The calculation equations of *E*_PICP_, *E*_*PINAW*_, and *E*_CWC_ are shown below.

27 $$E_{PICP} = \frac{1}{n}\sum\limits_{i = 1}^{n} {\tau_{i} }$$

28 $$E_{PINAW} = \frac{1}{n\delta }\sum\limits_{i = 1}^{n} {\left[ {U_{i} \left( {x_{i} } \right) - L_{i} \left( {x_{i} } \right)} \right]}$$

29 $$E_{CWC} = E_{PINAW} \left[ {1 + \gamma \left( {E_{PICP} } \right)e^{{ - \eta \left( {E_{PICP} - \mu } \right)}} } \right]$$

30 $$\gamma \left( {E_{PICP} } \right) = \left\{ \begin{gathered} 0,E_{PICP} \ge \mu \hfill \\ 1,E_{PICP} < \mu \hfill \\ \end{gathered} \right.$$

where: *n* represents the number of samples; *τ*_*i*_ is the Boolean quantity of the *ith* sample, if the predicted value falls within the prediction interval, then *τ*_*i*_ is 1, otherwise it is 0. *δ* is the range of the predicted target, which represents the difference between the maximum value and the minimum value, and is used for the normalization of *E*_PINAW_ . *U*(*x*_*i*_) and *L*(*x*_*i*_) are the upper and lower boundaries of the prediction interval, respectively. *x*_*i*_ is the input variable; *γ*(·) is the penalty coefficient. *η* is the confidence penalty coefficient of *E*_PIC*P*_. When *E*_PIC*P*_ does not satisfy the requirement of the confidence level *μ*, the composite indicator *E*_CWC_ will be amplified due to the amplification of the difference between the value of *E*_PIC*P*_ and *μ*, and when *E*_CWC_ meets the requirement of the confidence level *μ*, the value of *E*_CWC_ will be determined by the joint decision of *E*_PINAW_ and *η*.

### Analysis of the results of point forecasting

**Fig. 7:**
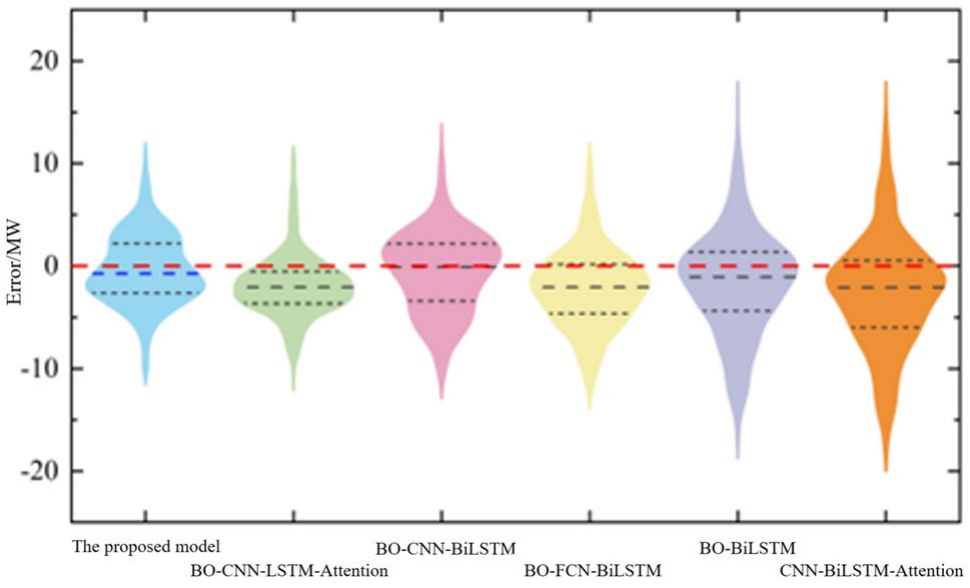
Comparison chart of prediction curves of three models

The dataset was divided into training, validation, and test sets according to a 7:2:1 ratio for single-step photovoltaic power prediction. To validate the effectiveness of the proposed BO-CNN-BiLSTM-Attention method for point prediction, five comparative models were constructed: BO-CNN-LSTM-Attention^45^, BO-CNN-BiLSTM^46^, BO-FCN-BiLSTM^47^, BO-BiLSTM^48^, and CNN-BiLSTM-Attention^49^. For fairness, Bayesian optimization was applied to models^45–49^, while only the CNN-BiLSTM-Attention model proposed in^49^ was retained to demonstrate the superiority of Bayesian optimization. The prediction performance metrics of each model on the test set are presented in Table 3, and the violin plots of prediction errors for each model on the test set are illustrated in Fig. 7.

**Table 3:** Predictive performance metrics of the model.

Model	RMSE (MW)	MAE (MW)
CNN-BiLSTM-attention	6.132	2.163
BO-BiLSTM	5.456	2.020
BO-FCN-BiLSTM	4.417	1.868
BO-CNN-BiLSTM	4.030	1.787
BO-CNN-LSTM-attention	3.641	1.706
BO-CNN-BiLSTM-attention	3.444	1.668

As shown in Table 3, the proposed BO-CNN-BiLSTM-Attention point prediction model achieves the best predictive performance, outperforming other models from the literature^45–49^ in both the Mean Absolute Error (MAE), which reflects overall error levels, and the Root Mean Square Error (RMSE), which captures extreme values. Compared to the CNN-LSTM-Attention model employed in^45^, the present study utilizes BiLSTM as the nonlinear fitting model, which better captures bidirectional temporal dependencies in photovoltaic power. Therefore, under the premise that both CNN-LSTM-Attention and the CNN-BiLSTM-Attention used in this study are optimized through Bayesian optimization, the hybrid model employed in this work still achieves superior predictive performance. Furthermore, the adoption of the Temporal Pattern Attention (TPA) mechanism enables the proposed model to learn more complex and detailed time series patterns. Consequently, compared to the CNN-BiLSTM model in^46^, even when Bayesian-based hyperparameter optimization is also applied, the proposed model demonstrates significantly improved prediction accuracy. It can also be observed that both the prediction accuracy and overall error level of the proposed model substantially outperform the CNN-BiLSTM-Attention model presented in^49^, thereby highlighting the importance of Bayesian optimization algorithms for enhancing the prediction accuracy of deep learning ensemble forecasting models. Finally, as intuitively shown in Fig. 7, the prediction errors of the proposed model are predominantly concentrated around zero, indicating that the overall prediction error level of the proposed method is relatively low. Moreover, the proposed point prediction model exhibits smaller extreme outliers compared to the other five models. In summary, the superiority of the proposed point prediction model for photovoltaic power forecasting applications can be validated.

### Clustering of similar day data and construction of prediction intervals

**Fig. 8:**
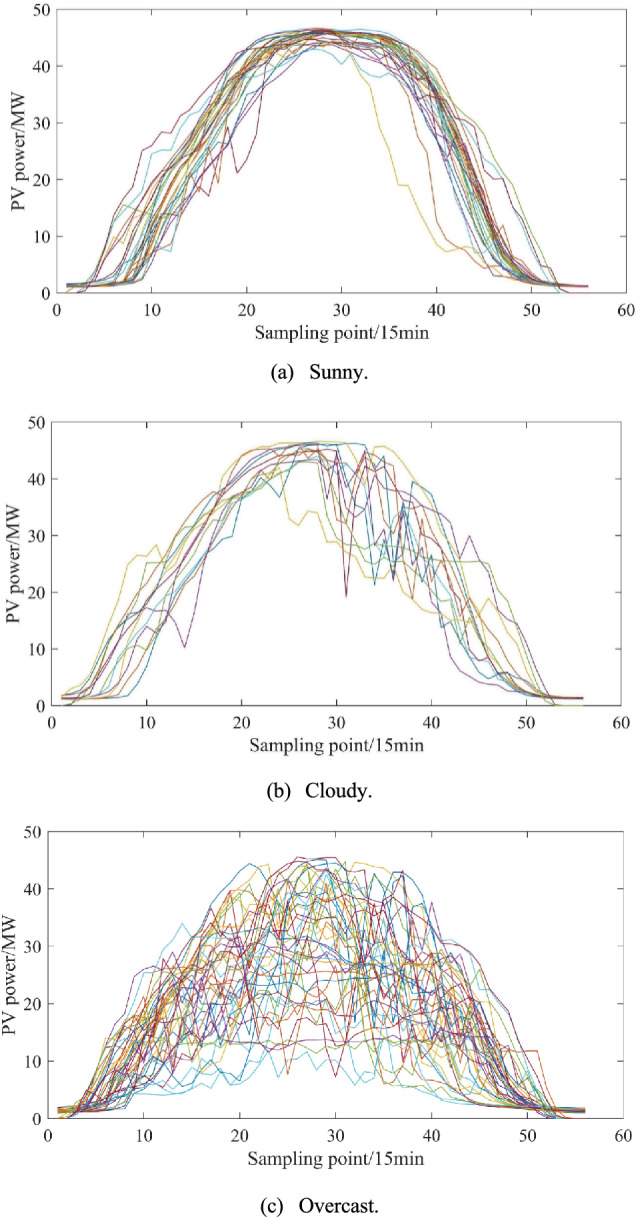
Similar day clustering results based on K-shape algorithm.

**Fig. 9:**
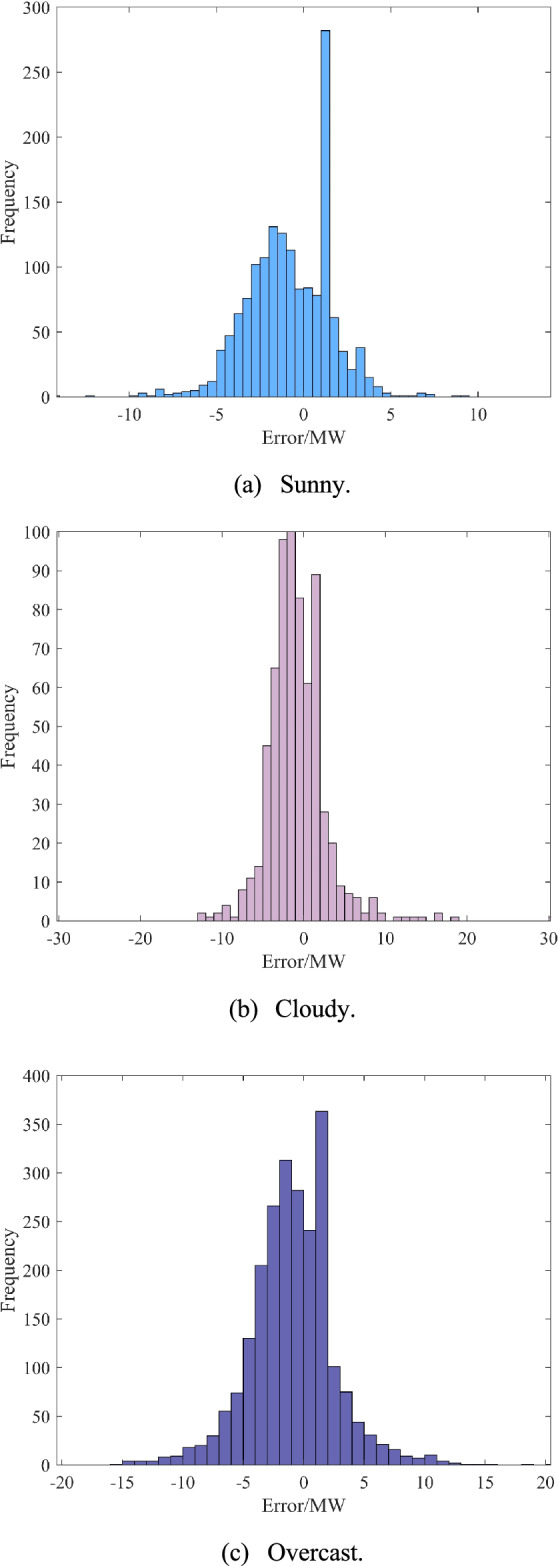
Histogram of the frequency distribution of prediction errors for different weather types.

To verify the effectiveness of the proposed interval construction method, this study conducts comprehensive validation experiments. The K-shape and ABKDE-based approach, which accounts for conditional dependence of prediction errors, is applied to three distinct weather scenarios: sunny, cloudy, and overcast conditions. For each weather type, one representative day is randomly selected for interval prediction analysis. The results are subsequently visualized and analyzed to assess method performance. The specific process is as follows: first, similar day data are matched for the time period to be predicted based on the K-shape clustering algorithm, as shown in Fig. 8. Then, the prediction error distribution of similar data clustering under different weather types is analyzed, and the histograms of prediction error distribution of similar data clustering under three weather types are given in Fig. 9. Finally, the ABKDE method is used to fit the histograms of the frequency distributions of different weather types to realize the interval prediction of the time period to be predicted.

### Analysis of interval forecast results

To validate the advantages and disadvantages of the proposed ABKDE method, in addition to the ABKDE approach employed in this study, the kernel density estimation (KDE) method proposed in^50^ and the Bootstrap method proposed in^51^ were used as comparative interval estimation methods, with confidence levels set at 90% and 95% for interval estimation. The interval estimation evaluation metrics for the three methods under different weather conditions are presented in Tables 4, 5 and 6, respectively.

**Table 4:** Evaluation metrics for various interval estimation methods on clear days.

Confidence level	Method	PICP	PINAW	CWC
95%	Bootstrap	0.8750	0.1950	8.4852
	KDE	0.9821	0.2749	0.2749
	ABKDE	0.9821	0.2726	0.2726
90%	Bootstrap	0.6964	0.1536	4045.3821
	KDE	0.8750	0.1985	0.8915
	ABKDE	0.9286	0.2097	0.2097

**Table 5:** Evaluation metrics for various interval estimation methods on cloudy days.

Confidence level	Method	PICP	PINAW	CWC
95%	Bootstrap	0.8929	0.2300	0.5587
	KDE	0.9643	0.3285	0.3285
	ABKDE	0.9643	0.3009	0.3009
90%	Bootstrap	0.8214	0.2192	11.3596
	KDE	0.8929	0.2420	0.5880
	ABKDE	0.8929	0.2315	0.5623

**Table 6:** Evaluation indicators of various interval estimation methods for overcast days.

Confidence level	Method	PICP	PINAW	CWC
95%	Bootstrap	0.9286	0.3254	1.2754
	KDE	0.9464	0.3737	0.8204
	ABKDE	0.9464	0.3701	0.8125
90%	Bootstrap	0.8571	0.2482	2.3642
	KDE	0.8750	0.2731	1.2264
	ABKDE	0.8929	0.2852	0.6928

**Fig. 10:**
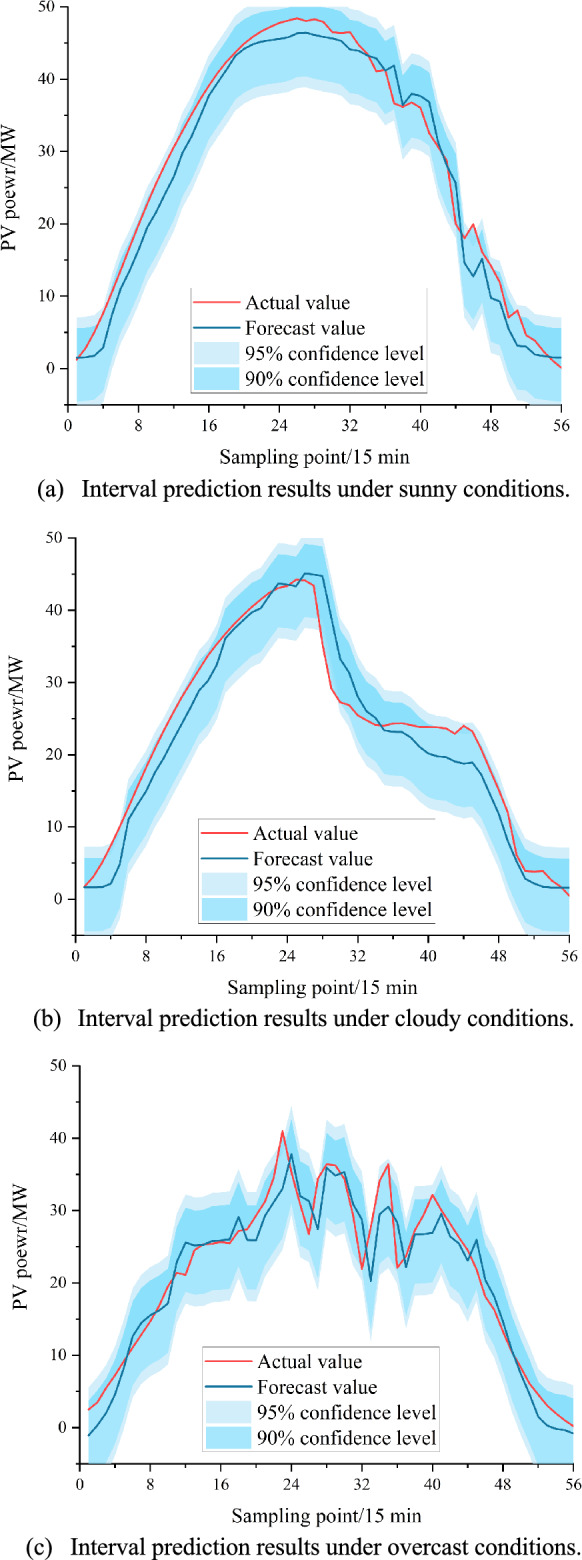
ABKDE-based interval estimation results

Overall, the iterative convergence-based ABKDE interval estimation method employed in this study outperforms the other two interval estimation methods across all three weather conditions, followed by the KDE method proposed in^46^, while the Bootstrap method proposed in^47^ demonstrates the poorest performance. From the perspective of weather type differences, interval estimation generally performs well under sunny conditions, moderately under cloudy conditions, and poorest under rainy conditions. As shown in Tables 4, 5 and 6, under sunny conditions, the ABKDE interval estimation accuracy reaches as high as 98.21% at the 95% confidence level and achieves 92.86% at the 90% confidence level. Under cloudy weather conditions, the accuracy is only 96.43% at the 95% confidence level, while under rainy weather conditions, it drops to as low as 94.64%. Figure 10 presents the interval estimation performance under different weather conditions achieved through the ABKDE method. As intuitively shown in Fig. 10, under sunny and cloudy weather conditions, the prediction intervals constructed by the proposed interval construction method at the 95% confidence level can essentially cover the vast majority of photovoltaic output points. However, under rainy conditions, due to significant variations in meteorological conditions that lead to rapid changes in photovoltaic power, the difficulty of interval prediction increases substantially, with photovoltaic output points that cannot be captured by the prediction interval at the 90% confidence level exceeding 10%. Nevertheless, at the 95% confidence level, most photovoltaic output points can still be covered, except for some points with rapid photovoltaic power fluctuations. In comparison, the interval prediction performance of KDE and Bootstrap methods is inferior to that of ABKDE, particularly the Bootstrap method. Although the average width of prediction intervals for Bootstrap is narrower than those of ABKDE and KDE, the interval coverage rate is very small, reaching only 0.9286 under the 95% confidence interval. In summary, the proposed iterative convergence-based ABKDE interval estimation method demonstrates superior predictive performance and can achieve effective interval prediction.

## Conclusion

Aiming at the problem of PV power volatility, stochasticity, and significant nonlinear features that make it difficult to predict accurately, this paper proposes a short-term PV power interval prediction method based on BO-CNN-BiLSTM-Attention taking into account the conditional dependence of the prediction error, and draws the following conclusions from the experiments:

1. The BO-CNN-BiLSTM-Attention hybrid neural network point prediction method fully combines the feature extraction capability of CNN, the nonlinear fitting capability of BiLSTM, and the TPA mechanism for the identification of complex time series patterns, and adopts an efficient Bayesian optimization algorithm to optimize the hyper-parameters of the combined prediction model, which can bring out the maximum performance of the combined prediction model and achieve better results than BO-FCN-BiLSTM, BO-CNN-BiLSTM and other models, and lays the foundation for the subsequent realization of accurate uncertainty prediction.
2. Considering the conditional dependence of the prediction error of PV power, this paper realizes the construction of future PV power prediction intervals based on the characteristics of the prediction error distribution of historical similar PV power data, and adopts the K-shape time series clustering algorithm to match the new predicted value of PV power for the time period to be predicted with historical similar PV power data to support the prediction of PV power intervals under different weather conditions.
3. The ABKDE method based on iterative convergence is used to realize the probability density estimation of PV power prediction error, which achieves better interval estimation than the KDE and Bootstrap methods, and is conducive to better capturing PV power output points.

## Data Availability

The datasets used and/or analysed during the current study available from the corresponding author on reasonable request.
